# Mapping the Comparative Effectiveness of Modified Constraint-Induced Movement Therapy, Hand-Arm Bimanual Intensive Training, and Mirror Therapy for Upper Extremity Rehabilitation in Children With Hemiplegic Cerebral Palsy: A Scoping Review

**DOI:** 10.7759/cureus.98440

**Published:** 2025-12-04

**Authors:** Rahul Bisen, Suvarna Ganvir

**Affiliations:** 1 Department of Neurophysiotherapy, Dr. Vithalrao Vikhe Patil Foundation's College of Physiotherapy, Ahilyanagar, IND

**Keywords:** hand-arm bimanual intensive training, hemiplegic cerebral palsy, mirror therapy, modified constraint-induced movement therapy, upper limb spasticity

## Abstract

Hemiplegic cerebral palsy (CP) usually impacts upper extremity function, leading to reduced participation and independence. Evidence-based treatment approaches such as modified constraint-induced movement therapy (mCIMT), hand-arm bimanual intensive training (HABIT), and mirror therapy have demonstrated neuroplasticity; however, direct comparisons among these interventions remain limited. Hence, this scoping review addresses evidence on the comparative efficacy, safety, and outcomes of these interventions in children with hemiplegic CP, mainly for the upper limb function. A literature search was performed in ScienceDirect, PubMed, and Google Scholar with specified keywords from January 2020 to September 2025. From 4,391 records, 14 high-quality studies rated using the Mixed Methods Appraisal Tool that fulfilled the eligibility criteria were included. The findings reported that all the interventions improved bimanual performance, upper limb motor control, and participation, with no adverse effects and short-term gains. mCIMT showed better unimanual outcomes; HABIT approach demonstrated superiority in bimanual coordination; mirror therapy enhanced dexterity/spasticity, especially as an adjunct. Comparative trials were absent, with heterogeneous protocols limiting rankings. Hence, mCIMT, HABIT, and mirror therapy offer safe, effective options for upper extremity rehabilitation in hemiplegic CP, supporting personalized/hybrid approaches. Future multicenter RCTs with standardized dosing and long-term follow-ups are needed to address gaps in comparisons and equity.

## Introduction and background

Cerebral palsy (CP) represents the leading cause of motor disability in childhood. The prevalence of CP ranges from 1.5 to 3 per 1,000 live births worldwide, arising from non-progressive disruptions in the developing brain that result in persistent impairments in movement, posture, and associated functions [1]. The hemiplegic CP, a subtype characterized by unilateral motor involvement, affects 20-30% of cases and often demonstrates pronounced asymmetry during usage of the limb [1,2]. Hemiplegic CP is usually more prevalent in low birth weight and preterm infants and develops from perinatal brain injuries [3]. Though recent trend illustrates a slight decrease in the prevalence of CP in high-income countries from approximately 2.1 to 1.6 per 1,000 live births due to advances in neonatal care, hemiplegic subtypes remain stable. Hence, this highlights barriers involved in early diagnosis and management [4].

An important impairment usually observed in children with hemiplegic CP is upper extremity dysfunction, which manifests as spasticity, reduced hand function, and sensory deficits. Upper extremity dysfunction impacts almost 80% of children and leads to compensatory overuse of the unaffected limb [5]. Additionally, upper extremity dysfunction is associated with impaired cognitive-motor integration during activities of daily living (ADLs), thereby limiting independence, social engagement, and participation in school [6,7]. During reach tasks, children with hemiplegic CP demonstrate increased elbow flexion and shoulder abduction, leading to pain, fatigue, and long-term orthopaedic complications that increase psychosocial burdens on families, which persist in adulthood as well [8,9]. Hence, to facilitate neuroplasticity, restore corticospinal pathways, and enhance coordination bimanually, early rehabilitation is essential [10].

Among various evidence-based methods, recent systematic evaluations in children with hemiplegic CP confirm modified constraint-induced movement therapy (mCIMT) efficacy in boosting upper limb function and grip strength, though benefits may vary by intervention dosage and adherence [11,12]. mCIMT modifies the original CIMT protocol by employing light resistance, shorter and more distributed session durations, alongside task-oriented, high-intensity unimanual practice to address learned non-use of the affected limb [13]. Moreover, in the paediatric population, hand-arm bimanual intensive training (HABIT) facilitates utilization of both upper limbs through repetitive, goal-directed bimanual activities, avoiding resistance for natural conscious integration and demonstrating sustained improvements in bimanual performance and daily functioning [14]. Additionally, a non-invasive approach that utilizes visual illusions to reflect movements of the unaffected limb is mirror therapy. This approach enhances motor planning, reduces spasticity, and improves dexterity when combined with conventional approaches by activating mirror neuron systems [15,16]. These approaches congruently lead to intensive, repetitive practice that facilitates neuroplasticity; however, individual applications report various protocols and outcomes.

Despite the beneficial advantages of these approaches, literature providing direct comparative analyses of mCIMT, HABIT, and mirror therapy (head-to-head trials) are limited [12-16]. The key limitations observed consist of the absence of trials evaluating differential impacts on long-term outcomes, optimal dosing regimens tailored to developmental stages, and potential adverse effects [12-16]. Moreover, integration with technology-based rehabilitation remains underexplored [17]. Hence, this scoping review addresses evidence on the comparative efficacy, safety, and outcomes of these interventions in children with hemiplegic CP.

## Review

The Preferred Reporting Items for Systematic reviews and Meta-Analyses extension for Scoping Reviews (PRISMA-ScR) guidelines [18] and the framework of Arksey and O’Malley (2005) were followed to synthesize findings for the current scoping review [19]. The methodology involved five stages, which are outlined below [20].

Stage 1: Identification of the research question

The primary research question was, “What is the current evidence on the comparative effectiveness and outcomes of mCIMT compared to HABIT and mirror therapy for improving upper extremity function in children with hemiplegic cerebral palsy?” The secondary objectives were to identify the differences in functional improvements between mCIMT, HABIT, and mirror therapy, to compare mCIMT, HABIT, and mirror therapy in terms of incidence and severity of adverse effects or limitations, and to identify gaps in evidence regarding optimal dosing regimens, direct intervention comparisons, and long-term child and family outcomes.

Stage 2: Identification of relevant studies

A comprehensive literature search was conducted in PubMed, Science Direct, and Google Scholar between January 2020 and September 2025. A combination of Medical Subject Headings (MeSH) terms and Boolean operators were used that consisted of (“modified constraint-induced movement therapy” OR “CIMT” OR “mCIMT”) OR (“hand-arm bimanual intensive training” OR “HABIT”) OR (“mirror therapy” OR “MT”) AND (“upper extremity” OR “upper limb”) AND (“cerebral palsy” OR “CP”) AND (“pediatric” OR “paediatric”). The search was limited to free full-text studies published in English. The search aimed to identify studies on the efficacy, safety, and outcomes of mCIMT, HABIT, and mirror therapy in improving upper extremity function in children with hemiplegic CP.

Stage 3: Selection of studies

The eligibility criteria for the studies to be included in the current review involved studies comparing or evaluating mCIMT, HABIT, and/or mirror therapy for upper extremity function, and studies reporting outcomes like motor function scores, quality of life, or functional participation. Additionally, studies including children (aged 0-18 years) with hemiplegic CP, studies published in English between January 2020 and September 2025 with full text availability, and original research studies were included. Whereas, review articles, opinion pieces, editorials, conference abstracts, non-peer-reviewed articles, studies lacking full text or relevance, or those in non-pediatric or non-CP settings were excluded. The population, concept, and context framework was P = children and adolescents (0-18 years) diagnosed with hemiplegic CP, C = efficacy and outcomes of mCIMT, HABIT, or mirror therapy, and C = pediatric rehabilitation and neurology settings globally. For the removal of duplicates and irrelevant studies, two reviewers independently screened titles and abstracts. The discrepancies between the reviewers were resolved through mutual discussion.

Stage 4: Data charting

The extracted data consisted of study characteristics, intervention details, findings on functional improvements, long-term outcomes, research gaps, limitations or adverse effects, and recommendations for future research. The Mixed Methods Appraisal Tool was used to assess the methodological quality of the included studies and classify the studies as high, moderate, or low quality based on the criteria mentioned. The tool is a widely used instrument for assessing qualitative, quantitative descriptive (cross-sectional), mixed methods studies, and nonrandomized and randomized controlled trials (RCTs) [21].

Stage 5: Collating, summarizing, and reporting the results

A narrative synthesis approach through figures, text, and tables was performed to summarize findings. The results compared mCIMT versus HABIT versus mirror therapy approaches to highlight the effectiveness of these techniques and associated outcomes in terms of functional improvements, adverse effects, and long-term outcomes. Moreover, the synthesis identified gaps, particularly in direct comparisons and long-term effects, and proposed recommendations for future research and clinical practice.

Results

**Figure 1:**
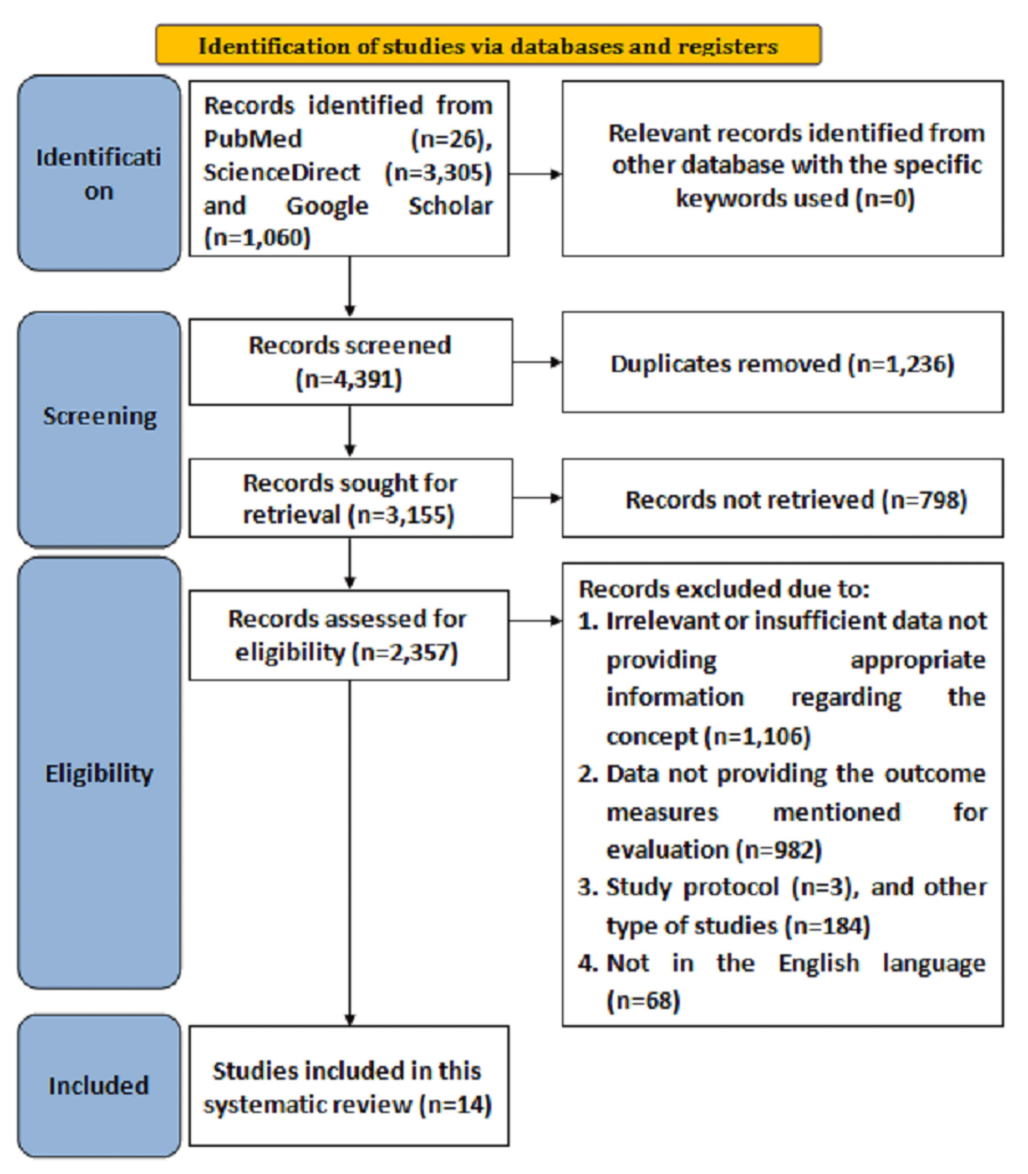
Preferred Reporting Items for Systematic Reviews and Meta-Analyses (PRISMA) search strategy

Initially, overall, 4,391 articles were found after searching the databases from 2020 to August 2025 with the specified keywords. Duplicate articles consisting of 1,236 were removed after screening, following which 3,155 articles were sought for retrieval, from which 798 articles were not retrieved based on title and abstract evaluation. Moreover, overall, 2,357 records were assessed for eligibility, from which 2,343 records were excluded due to irrelevant data not providing appropriate information regarding the concept (n =1,106), data not providing the outcome measures mentioned for evaluation (n = 982), study protocol (n = 3), other types of studies (n = 184), and not being in the English language (n = 68). Hence, for the current review, 14 studies fulfilling the eligibility criteria were included. The PRISMA flowchart reporting search strategy is illustrated in Figure 1.

The demographic characteristics and quality of the included studies are reported in Table 1.

**Table 1:** Demographic characteristics of the included studies RCT = Randomized controlled trial, AOT = Action-observation training, mCIMT = Modified constraint-induced movement therapy, GMFCS = Gross motor function classification system, CFCS = Communication function classification system

Author and year	Study design	Location	Sample size	Study population	Intervention	Quality of the studies
Simon-Martinez et al. (2020) [22]	RCT	KU Leuven, Belgium	36	Children with unilateral cerebral palsy (uCP), aged 6-12 years, with House Functional Classification scale scores between 4 (poor active assist) and 8 (spontaneous use), sufficient cooperation to complete testing; mean age 9 years 6 months, 27 boys, Manual Ability Classification System levels I-III.	mCIMT + AOT: 9-day mCIMT camp wearing a splint on the less impaired hand for 6 h/day (total 54 h therapy) + 15 h AOT (video-observation and execution of unimanual tasks). mCIMT + placebo: same mCIMT camp + watching biological-motion free videos and executing tasks.	High
Mohamed et al. (2021) [23]	RCT	Cairo, Egypt	60	Children with hemiplegic cerebral palsy, aged 6-8 years, with grade 1 or 1+ spasticity, able to understand instructions, and no severe cognitive impairments or visual deficits.	All groups underwent a 1-hour upper limb exercise program, 5 days/week for 12 weeks, plus 1-hour routine physical therapy. Group A: Program with mirror therapy (MT) and taping (Kinesio tape on the affected upper limb). Group B: Program with modified constraint-induced movement therapy (mCIMT, unaffected limb restrained). Group C: Program with MT alone.	High
Friel et al. (2021) [24]	RCT	University laboratory settings in New York, USA, and Brussels, Belgium	82	Children with unilateral spastic cerebral palsy (USCP), aged 5 years 10 months to 17 years, with varying corticospinal tract (CST) connectivity patterns (contralateral, ipsilateral, or bilateral), stratified by age, sex, baseline hand function, and CST pattern.	Constraint-Induced Movement Therapy (CIMT) or Bimanual training, both delivered in a day-camp setting for 90 hours (specific dosage not detailed), with randomization stratified by CST connectivity, age, sex, and baseline hand function.	High
Madbouly et al. (2021) [25]	RCT	Cairo and Alexandria, Egypt	40	Children with right hemiparetic cerebral palsy, aged 5-8 years, grade 1+ spasticity, level III Manual Ability Classification System (MACS), no severe cognitive or visual impairments.	Group A (control): Modified Constraint-Induced Movement Therapy (mCIMT) for 3 hours/day, 5 days/week for 4 weeks, with the unaffected hand restrained using a sling. Group B: Mirror therapy for 30 minutes/day, 5 days/week for 4 weeks, using a mirror box to reflect movements of the unaffected hand. Both groups received regular intensive physical and occupational therapy.	High
Preetha et al. (2021) [26]	RCT	Chennai, Tamil Nadu, India	30	Patients with subacute cerebrovascular accident (stroke) resulting in hemiplegia, Mini-Mental State Examination score >21, mild to moderate motor impairment (levels 3-5 on functional test for hemiplegic upper extremity), middle cerebral artery affected, mild to moderate spasticity, able to perform active wrist extension >10° and thumb abduction >10°	Constraint-Induced Movement Therapy (CIMT) group: 4 weeks of therapy with the unaffected arm restrained using a sling or splint, performing tasks with pebbles, smiley balls, coins, and a pegboard. Task-Based Mirror Therapy (TBMT) group: 4 weeks of therapy using a mirror to reflect movements of the unaffected arm, performing similar tasks. Specific dosage (hours/day) is not detailed.	High
Bingöl et al. (2022) [27]	RCT	Mus, Turkey	32	Children with hemiplegic cerebral palsy, mean age 10.43±2.9 years, 15 girls, 17 boys, functional levels I-III (MACS, GMFCS, CFCS), mainstreamed in regular school.	mCIMT: 10 weeks, 3 days/week, 2.5 hours/day (face-to-face in school, home, and clinical environments plus home practice). BIT: 10 weeks, 3 days/week, 2.5 hours/day (face-to-face in school, home, and clinical environments plus home practice).	High
Jobst et al. (2022) [28]	Prospective cohort study	Toronto, Ontario, Canada	12	Children with hemiplegic cerebral palsy (HCP), mean age 7.5±2.4 years, aged 5.0-12.9 years, 9 males and 3 females, 10 with right-hand affected and 2 with left-hand affected.	Somatosensory enhanced CIMT camp: 3-week program including nonremovable below-elbow cast on unaffected limb for the first week (24 hours/day), followed by a 2-week day camp with removable cast worn part-time (4 hours/day, 5 days/week, total 40 hours); focused on unilateral and bilateral motor/somatosensory activities, with enhanced somatosensory components targeting tactile, stereognosis, and proprioceptive modalities.	High
Araneda et al. (2022) [29]	RCT	Brussels, Belgium	31	Children with unilateral cerebral palsy, mean age 9 years 4 months±4 years 3 months, range 5 years 4 months to 17 years 3 months; 14 females, 17 males; inclusion: school attainment equal to typically developing peers, able to follow instructions; exclusion: uncontrollable seizures, recent/planned botulinum toxin or surgery, visual problems.	Treatment group: 90 hours of Hand and Arm Bimanual Intensive Therapy Including Lower Extremity (HABIT-ILE) over 2 weeks (9 hours/day, 10 weekdays), focusing on bimanual activities with increasing difficulty and five daily life goals; control group: customary treatment (1-5 hours/week physiotherapy/occupational therapy).	High
Palomo-Carrión et al. (2023) [30]	RCT	Two public hospitals and an infantile hemiplegia association in Spain (Toledo and Salamanca regions).	21	Children with congenital hemiplegia, aged 5-8 years, with low/very low bimanual functional performance, Assisting Hand Assessment (AHA) < 40 units); Manual Ability Classification System levels I-III; no prior upper limb surgery or botulinum toxin in the last 6 months; able to follow instructions.	Both groups received 100 hours of intensive upper limb therapies (2 hours/day, 5 days/week, 10 weeks). Experimental group (n=11): 80 hours modified constraint-induced movement therapy (mCIMT) first (weeks 1-8) + 20 hours bimanual intensive therapy (BIT) (weeks 9-10). Control group (n=10): 80 hours BIT first (weeks 1-8) + 20 hours mCIMT (weeks 9-10). mCIMT involved the restraint of the unaffected limb with a sling and intensive unimanual tasks; BIT focused on bimanual coordination tasks.	High
Shih et al. (2023) [31]	RCT	Taipei, Taiwan	29	Children with unilateral cerebral palsy (UCP), aged 5-12 years, with congenital UCP, considerable nonuse of the affected upper extremity (amount-of-use score on Revised Pediatric Motor Activity Log.	Kinect-based CIMT: 2.25 h/day, 2 days/week for 8 weeks (total 36 h), using Kinect 2 sensor with games "Adventure Island" and "Kitten Island" in natural environments, supervised by therapists, with contextual restraint and pause mechanism. Therapist-based CIMT: Same dosage, delivered by therapists with family assistance in natural environments, using age-appropriate therapeutic games with gentle restraint (verbal/physical guidance).	High
Bhargavi et al. (2023) [32]	RCT	GSL College of Physiotherapy, GSL Medical College, Rajamahendravaram, Andhra Pradesh, India.	64	Children with unilateral cerebral palsy	Group I (n=32): Constraint-Induced Movement Therapy (CIMT) for 5 sessions per week for 4 weeks, with restraint on the less-affected limb to promote use of the affected upper extremity. Group II (n=32): Bimanual Training for 5 sessions per week for 4 weeks, focusing on coordinated use of both upper limbs. Specific session duration not specified.	High
Abdel Ghafar et al. (2025) [33]	RCT	Nearby pediatric rehabilitation facilities (specific locations not detailed; affiliations suggest Saudi Arabia and Egypt).	50	Children with unilateral cerebral palsy (UCP), aged 5–9 years, including 27 boys and 25 girls (final analysis: 50 children); inclusion criteria: diagnosis of congenital UCP, hypertonia grade 1 or 1+ on the modified Ashworth scale, level II or III manual ability according to the Manual Ability Classification System (MACS), ability to comprehend and follow instructions; exclusion criteria: severe orthopedic dysfunction (e.g., severe fixed hand deformity), cognitive impairment, poor verbal and visual acuity, uncontrolled seizures, prior hand surgery.	MVF: 30 min sessions, 5 times per week, over 12 weeks, involving mirror visual feedback with fine motor exercises (e.g., rolling a ball, moving beads, squeezing a stress ball, stretching rubber bands, rotating pencils, each 10 times with 20s rest between exercises) + traditional physical therapy (45–60 min, 3 times per week, 12 weeks), including functional unilateral upper extremity training and fine motor exercises; mCIMT: 30 min sessions, 5 times per week, over 12 weeks, involving functional tasks with the affected hand, unaffected hand restrained by an arm sling, including activities like dough activities, bottle and marble activities, and manual chores + same traditional physical therapy.	High
Ilyas et al. (2025) [34]	RCT	Rising Sun Institute for Special Children, Lahore, Pakistan	30	Children with spastic hemiplegic cerebral palsy, aged 4–9 years, Manual Ability Classification System (MACS) levels I–III, no severe cognitive impairments, able to follow instructions.	CIMT group: 6 hours/day of modified Constraint-Induced Movement Therapy (mCIMT) for 3 weeks, with the unaffected limb restrained using a mitt, focusing on unimanual tasks. HABIT group: 6 hours/day of Hand–Arm Bimanual Intensive Training (HABIT) for 3 weeks, focusing on bimanual coordination tasks. Both groups received standardized physiotherapy alongside their respective therapies.	High
Boyd et al. (2025) [35]	RCT	Australia (Queensland, New South Wales, Victoria, Western Australia) and the United States (Ohio, Minnesota)	96	Infants at high risk of unilateral cerebral palsy, aged 3-9 months corrected age (mean 6.5±1.6 months), with asymmetric brain lesion on neuroimaging, absent fidgety General Movements Assessment, Hammersmith Infant Neurological Examination scores below cerebral palsy cut-points, and >3-point difference between hands on Hand Assessment Infants (HAI) congruent with neuroimaging; 51 males (53%), 52 with right hemiplegia (54%), median gestation 37 weeks.	Baby-CIMT (modified constraint-induced movement therapy with soft restraint on less-impaired limb and intensive training of hemiplegic arm) versus Baby-BIM (bimanual therapy encouraging use of both upper limbs together); home-based, 6-9 months duration, daily dose varying by age from 20-40 minutes (total 70-89.2 hours), with bimonthly therapist home visits and virtual sessions; four ability levels based on HAI scores, incorporating parent coaching on emotional availability and mental health support.	High

Moreover, the various findings observed in outcome measures, research gaps, and recommendations for improving future practice are reported in Table 2.

**Table 2:** Summary of the included studies AOT = Action-observation training, mCIMT = Modified constraint-induced movement therapy, RU = Reaching upward, RGV = Reach-to-grasp a vertically oriented cylinder, HTS = Hand-to-shoulder, MT = Mirror therapy, CST = Cortico spinal tract, TBMT = Task-based mirror therapy, BIT =  Bimanual training, SEF = Somatosensory evoked fields, PPV = Percentage of movement time when peak velocity occurred, UCP = Unilateral cerebral palsy, MVF = Mirror visual feedback, EA-SR = Emotional availability-self report, BIM = Bimanual therapy, HAI = Hand assessment infants, MD = Mean difference, MRI = Magnetic resonance imaging

Author and year	Findings observed in outcome measures	Adverse effects or limitations reported	Research gaps	Recommendations for future research
Simon-Martinez et al. (2020) [22]	Adding AOT to mCIMT mainly affected movement duration during reaching upwards task at 6 months follow-up (p=0.001, d=0.64); mCIMT (with or without AOT) improved peak velocity (RU p=0.01, RGV p=0.008), trajectory straightness (HTS p=0.04), and proximal movement patterns (trunk, scapula, shoulder); poor relation between clinical (muscle tone, strength) and kinematic improvements (low-moderate correlations); no distal improvements at elbow/wrist; no adverse effects reported.	No adverse effects; limitations include a smaller sample than planned (36 versus 44) due to missing data, high individual variability, tasks not challenging enough, and exclusion of children with poor cooperation or recent treatments.	Mechanisms of AOT combined with mCIMT; role of mirror neuron system in children with different brain lesions; individual factors influencing response (e.g., lesion type, age).	Include larger samples; explore individual predictors (brain lesion, age); use more challenging tasks; investigate neural mechanisms with neuroimaging.
Mohamed et al. (2021) [23]	All groups showed significant improvements post-treatment in Quality of Upper Extremity Skills Test (QUEST), Box and Block Test (BBT), and grip strength (hand-held dynamometer); Group A (MT + taping) demonstrated superior improvements compared to Group B (mCIMT) and Group C (MT alone) (specific p-values and effect sizes not provided in abstract).	No adverse effects reported; limitations include lack of long-term follow-up, no blinding details, and focus on a narrow age range (6-8 years).	Long-term effects of MT with taping, mCIMT, and MT alone; optimal dosing and combination strategies; applicability to broader age groups or severity levels; lack of comparison with other therapies (e.g., bimanual training).	Conduct studies with longer follow-up to assess sustained outcomes; explore optimal dosing and combination protocols; include diverse age groups and severity levels; compare with other interventions like bimanual training.
Friel et al. (2021) [24]	Both CIMT and Bimanual training groups showed statistically similar improvements across all measures (Jebsen-Taylor Test of Hand Function, Assisting Hand Assessment, Box and Block Test, ABILHAND-Kids, Canadian Occupational Performance Measure, Pediatric Evaluation of Disability Inventory) regardless of CST connectivity pattern (p< 0.05 for all); improvements sustained at 6-month follow-up; no significant differences between groups or CST types.	No adverse effects reported; limitations include lack of a control group, potential bias from self-selected participants, and variability in intervention delivery across sites; small sample size per CST subgroup limiting power for subgroup analyses.	Unclear long-term effects beyond 6 months; role of CST reorganization timing in treatment response; optimal intensity/duration of interventions; lack of comparison with no-treatment controls.	Conduct long-term follow-up studies; explore timing of CST reorganization and its impact; test varying intensities/durations of CIMT and Bimanual training; include no-treatment control groups to assess natural progression.
Madbouly et al. (2021) [25]	Significant increase in Quality of Upper Extremity Skills Test (QUEST) scores for Group A post-treatment compared to Group B (p=0.0001); mCIMT showed greater effectiveness in improving affected hand functions.	No adverse effects reported; limitations include small sample size, focus on right hemiparesis only, short duration (4 weeks), lack of long-term follow-up, and no blinding of therapists or participants.	Limited data on long-term effects; lack of comparison with other therapies; unclear optimal duration and intensity; applicability to different MACS levels or bilateral cases.	Conduct studies with longer follow-up periods; compare mCIMT and mirror therapy with other interventions; explore optimal dosing and intensity; include diverse populations (e.g., different MACS levels, bilateral cases).
Preetha et al. (2021) [26]	CIMT group showed greater improvement in Fugl-Meyer Motor Function Assessment (pre-test mean 6.3±1.45; post-test mean 11.13±1.36) compared to TBMT group (pre-test mean 5.733±1.67; post-test mean 9.86±1.92); specific p-value not reported, but CIMT deemed more effective; no adverse effects mentioned.	No adverse effects reported; limitations include small sample size, short intervention duration (4 weeks), no long-term follow-up, lack of blinding details, and focus on stroke patients rather than cerebral palsy, which may limit applicability to the review context.	Long-term effects of CIMT and TBMT; optimal dosing and intensity; applicability to cerebral palsy populations; inclusion of broader outcome measures (e.g., quality of life, bimanual function); comparison with other interventions.	Conduct studies with longer follow-up to assess sustained outcomes; optimize intervention duration and intensity; include cerebral palsy populations; incorporate broader outcomes like quality of life and bimanual function; compare with other therapies (e.g., bimanual training).
Bingöl et al. (2022) [27]	mCIMT resulted in more significant improvements in upper extremity body function (grip strength via handheld dynamometer), activity through Quality of Upper Extremity Skills Test (QUEST) dissociated movements and grasp subtests, Children’s Hand-use Experience Questionnaire (CHEQ), ABILHAND-Kids, Pediatric Upper Extremity Motor Activity Log (PUMAL), and participation through Child and Adolescent Scale of Participation (CASP) immediately post-intervention compared to BIT, with large effect sizes (dmCIMT > dBIT); improvements maintained at 16 weeks post-intervention in mCIMT group; BIT showed moderate improvements in activity and participation but no significant change in grip strength; no adverse events related to restraint material or activity training reported; all children completed interventions.	No adverse events; limitations include a small sample size preventing subgroup analysis, no control group (e.g., usual care), short follow-up (16 weeks), single-center design, and potential bias from non-blinded interventionist (though assessor blinded).	Optimal dosing regimens have not been explored in school settings; no direct comparison with mirror therapy; long-term outcomes limited to 16 weeks; limited understanding of transfer from unimanual to bimanual skills; need for larger target populations to enable subgroup analyses.	Conduct multicenter RCTs to test varying durations and intensities of mCIMT and BIT in school-based settings, including mirror therapy arms, and extend follow-up to 6-12 months to assess sustained quality of life and functional participation; include larger samples for subgroup analyses; incorporate control groups for comparison to usual care.
Jobst et al. (2022) [28]	Significant improvements in tactile registration for the affected hand (Z=2.39, P=0.02); statistically and clinically significant improvements in QUEST total (t=3.24, P=0.007), QUEST grasp (t=3.24, P=0.007), Assisting Hand Assessment (Z=2.25, P=0.03), and Jebsen-Taylor Hand Function Test (t=-2.62, P=0.03); significant increase in SEF peak amplitude in the affected hand at 100 ms post-stimulus (t=-2.22, P=0.04); no changes in other somatosensory measures (2-point discrimination, stereognosis, proprioception, kinesthesia) or grip strength; child satisfaction, quality of life, and long-term functional outcomes not assessed.	No adverse effects reported; limitations include small sample size (n=12, with neuroimaging data from n=10), lack of control group, potential for practice effects on repeated assessments, and short-term follow-up (1 week post-intervention).	Limited understanding of CIMT's independent effects on somatosensory function; need for larger samples to confirm findings; absence of controlled designs to isolate CIMT's impact; lack of long-term follow-up; no direct comparison with HABIT or mirror therapy; unclear neural mechanisms beyond SEF amplitude changes.	Evaluate the effectiveness of sensory-enhanced CIMT in larger samples and controlled study designs; include longer-term follow-up to assess sustained outcomes; incorporate comparisons with other interventions like HABIT or mirror therapy; explore additional neuroimaging metrics to understand brain reorganization.
Araneda et al. (2022) [29]	Significant decrease in mirror movements in more-affected hand (mean difference 0.97, 95% CI 0.51–1.42, p<0.001) and less-affected hand (mean difference 0.71, 95% CI 0.37–1.0, p< 0.001) post-HABIT-ILE, maintained at 3 months; inverse correlations with daily living changes, especially less-affected hand (e.g., Assisting Hand Assessment rs=-0.54, p=0.002); significant improvements in Assisting Hand Assessment, Pediatric Evaluation of Disability Inventory, Canadian Occupational Performance Measure in treatment group (p< 0.001 for most).	No adverse effects; limitations: mostly Manual Ability Classification System level II participants, limiting generalizability; Woods and Teuber Scale reliability noted, but less sensitive than grip force measures.	Unexplored changes in interhemispheric connections; inconclusive impact of corticospinal tract types due to sample size; limited to a narrower Manual Ability Classification System range.	Examine interhemispheric connection changes; include broader Manual Ability Classification System levels; use more sensitive tools like grip force measures.
Palomo-Carrión et al. (2023) [30]	Experimental group: bimanual functional performance increased 22 AHA units at week 8 (after mCIMT) vs. control's 3.7 units (after BIT); at week 10, experimental total +27.3 units, control +14.3 units (control gained 10.6 units after mCIMT). Quality of life through Pediatric Quality of Life Inventory Cerebral-Palsy module (PedsQL CP): greatest gains after mCIMT (13.1 points in experimental at week 8, 13.7 in control at week 10); sustained at 10 weeks. No significant between-group differences at week 10, but order affected magnitude (mCIMT first yielded larger bimanual gains).	No adverse effects; high adherence, no dropouts. Limitations: small sample size (limits generalizability and subgroup analysis); single-blinded (assessors blinded, but children/parents not); no long-term follow-up beyond 10 weeks; specific to children with low bimanual performance; potential bias from non-blinded participants.	Optimal order and dosing in hybrid mCIMT/BIT protocols for children with low bimanual performance; long-term sustainability of gains; effects in larger, more diverse samples; influence of cognitive/motivational factors.	Conduct larger multicenter RCTs with extended follow-up (e.g., 6-12 months) to assess sustained outcomes; explore hybrid protocol variations in dosing/order; include broader populations (e.g., different ages, severities) and control for cognitive factors; incorporate neuroimaging to understand mechanisms.
Shih et al. (2023) [31]	Both groups showed similar improvements in upper extremity motor control (reaction time, movement time, peak velocity, percentage of movement time to peak velocity, movement units) and daily motor function (amount of use and quality of use on Revised Pediatric Motor Activity Log); kinect-based CIMT group showed greater improvements in trunk motor control (greater normalized endpoint displacement during PPV before phase, smaller normalized trunk displacement during PPV after phase, greater trunk contribution slope in both phases) compared to therapist-based CIMT (F>4.862, p<0.036).	No adverse effects reported; limitations include a small sample size limiting generalizability, a homogeneous population, no follow-up assessments for long-term effects, and Kinect-based CIMT limited to two games, potentially reducing variety.	Limited understanding of long-term effects; effects in larger, more diverse samples; need for more varied Kinect-based games; potential of Kinect-based CIMT via telerehabilitation.	Conduct studies with larger, diverse samples; include follow-up assessments for long-term effects; develop more games based on CIMT principles; explore Kinect-based CIMT through telerehabilitation.
Bhargavi et al. (2023) [32]	Both groups showed improvements in all parameters of the Jebsen-Taylor Hand Function Test (JTHFT) based on pre-test, intermediate test, and post-test scores; the CIMT group demonstrated statistically significant greater improvement compared to the Bimanual Training group (p-value not specified in abstract but noted as significant); no specific data on effect sizes or clinical significance was provided.	No adverse effects reported; limitations include lack of detailed demographic information (e.g., age range, severity levels), no mention of blinding, short intervention duration (4 weeks), and no follow-up beyond immediate post-intervention assessment.	Limited information on long-term effects; unclear optimal duration and intensity of interventions; lack of comparison with other therapies (e.g., mirror therapy); need for broader demographic inclusion (e.g., age, severity); absence of quality of life or participation outcomes.	Conduct studies with longer follow-up periods to assess sustained outcomes; explore optimal dosing and intensity for CIMT and Bimanual Training; include comparisons with other interventions like mirror therapy; incorporate broader outcome measures such as quality of life and participation; expand to diverse populations with detailed demographic reporting.
Abdel Ghafar et al. (2025) [33]	Significant enhancements in manual dexterity through Box and Block Test (BBT) and maximum isometric hand strength (hydraulic handheld dynamometer) in both groups post-intervention; mCIMT group showed significantly greater gains: BBT scores (p = 0.014, effect size 0.75), maximum isometric hand strength (p = 0.017, effect size 0.79); within-group comparisons: mCIMT group (p = 0.001 for BBT, p = 0.012 for strength), MVF group (p = 0.002 for BBT, p = 0.001 for strength); pre-intervention, no significant between-group differences (p = 0.672 for BBT, p = 0.375 for strength).	No adverse effects explicitly reported; limitations: variability in children’s ability to understand and carry out tasks, particularly in the MVF group, influenced by attention span and comprehension; findings specific to children aged 5–9 years with UCP, may not apply to younger/older children or different impairment severities; assessments required active participation, potentially introducing variability due to motivation, fatigue, or comprehension.	Limitations of previous research, such as small sample sizes, short-term technique application, varying treatment durations, and failure to specifically consider hand function as an essential outcome, need for studies with extended duration and equated treatment durations to provide clear evidence.	Explore combining mCIMT with MVF or other complementary therapies to potentially enhance benefits, and consider individual factors like severity of impairment, patient motivation, and treatment access; consider applicability to different age groups and impairment severities.
Ilyas et al. (2025) [34]	Both groups showed significant within-group improvements (p< 0.001) on the Assisting Hand Assessment (AHA) and Jebsen–Taylor Test of Hand Function (JTTHF). Between-group comparisons favored CIMT: greater AHA gains post-intervention (mean difference 6.7, p=0.001) and at 6 months (7.3, p< 0.001); greater JTTHF improvements post-intervention (mean difference 2.2, p=0.004) and at 6 months (3.7, p< 0.001). Large effect sizes observed for CIMT.	No adverse effects reported; limitations include small sample size, lack of blinding, short intervention duration (3 weeks), and potential variability in therapist delivery in a low-resource setting.	Limited data on long-term effects beyond 6 months; optimal dosing and duration of CIMT and HABIT; lack of hybrid model comparisons; applicability in diverse socioeconomic contexts.	Conduct studies with longer follow-up periods; explore optimal therapy durations and intensities; investigate hybrid CIMT-HABIT models; expand research to diverse global settings with varying resource levels.
Boyd et al. (2025) [35]	No significant between-group differences in primary outcomes (HAI units: MD 0.98, 95% CI -0.94 to 2.91, P=.31; HAI impaired EaHS: MD 0.54, 95% CI -1.24 to 2.32, P=.55) post-intervention. Both groups (Baby-CIMT and Baby-BIM) showed significant improvements from baseline in HAI units (Baby-CIMT MD 15.9, P6 months. At 24 months, 67% diagnosed with UCP (35 Baby-CIMT, 29 Baby-BIM). Both groups improved child involvement on EA-SR (Baby-CIMT MD 2.9, P=.001; Baby-BIM MD 3.3, P<0.001); Baby-BIM reduced parental depression (MD -1.1, P=.04). No adverse events; high intervention fidelity (median 16/16) and parent engagement (median 82/84); home practice averaged 44.6±22.6 hours.	No adverse events in either group; limitations include smaller sample size than planned (96/144) due to COVID-19, potentially reducing power to detect smaller differences; no true control group (considered unethical); HAI not validated beyond 12 months corrected age (though used up to 15 months); potential therapist contamination risk (mitigated by fidelity checks); reliance on parent-reported home practice diaries; lack of follow-up MRI to assess brain lesion evolution; attrition 12% (no differences between completers and dropouts).	Limited understanding of differential impacts on brain reorganization (e.g., corticospinal and thalamocortical pathways); need for studies starting < 4 months corrected age; long-term outcomes beyond 24 months (e.g., 4-6 years); hybrid CIMT/BIM approaches; accurate early identification using MRI, General Movements Assessment, and Hammersmith Infant Neurological Examination; factors influencing choice between unimanual vs bimanual therapy.	Use early MRI, Motor Optimality Score-Revised asymmetries, and Hammersmith Infant Neurological Examination (>5 asymmetries) for identification; explore hybrid Baby-CIMT/Baby-BIM in adaptive designs; conduct longer-term follow-up to 4-5 years; incorporate advanced MRI (e.g., diffusion techniques, cortical thickness) for brain structure-function relationships; focus on earlier commencement (<4 months) to optimize neuroplasticity; evaluate in larger samples with full planned power.

Discussion

This scoping review involved 14 high-quality RCTs and prospective cohort studies to summarize evidence regarding comparative effectiveness and outcomes of mCIMT, HABIT, or bimanual intensive therapy (BIT), and mirror therapy approaches. The review encompassed 652 children, primarily classified at Manual Ability Classification System (MACS) levels I-III, aged three months to 17 years, with hemiplegic or unilateral CP. All the approaches reported improvements in upper extremity function consistently, including motor control, bimanual performance, and participation in ADLs. The results demonstrated that in comparison to HABIT and mirror therapy approaches, mCIMT showed enhanced unimanual outcomes (dissociated movements and grasp quality) with large effect sizes. Mirror therapy, when combined with taping, reduced spasticity and enhanced dexterity, whereas HABIT/BIT reported effectiveness in bimanual coordination. Additionally, the improvements gained through all the interventions were sustained up to short-term follow-ups, and the children showed no adverse effects, though the studies lacked direct comparisons of all three approaches, limiting definitive conclusions regarding relative effectiveness among these techniques.

A previous study by Sakzewski reported that mCIMT performed in hemiplegic CP children aged 3.5-10 years significantly improved upper limb function by enhancing unimanual grip strength and function, thereby supporting the findings of the current review, which reported moderate to large effect sizes. Hence, the intensive unimanual practice overcame the learned non-use [36]. Similarly, Psychouli and Kennedy noted that home-based protocols for the CIMT approach have shown improvements in the peak velocity and reach trajectory, reinforcing kinematic enhancements [37]. Moreover, Gardas et al. demonstrated that in children with unilateral CP, the HABIT approach enhances ADLs and social participation in real-world situations by improving hand function [38]. A systematic review and meta-analysis further highlighted effectiveness of mirror therapy children with hemiplegic CP in improving dexterity, and motor planning via mirror neuron activation [17]. These techniques illustrate facilitation of neuroplasticity, particularly during the early rehabilitation period.

However, a study by Sakzewski et al. reported equal enhancements in unimanual and bimanual functions from CIMT and BIT approaches, along with sustained effects at one-year follow-up [39]. Similarly, a Cochrane review by Tervahauta et al. illustrated that neither CIMT nor BIT in children with unilateral CP could demonstrate overall effectiveness, due to differences in effect sizes and various protocol regimens employed in improving hand functions [40]. Though mirror therapy has reported beneficial effects in addition to conventional approaches [23,25,33], a study by Yoon et al. reported similar outcomes for mirror therapy and mCIMT in stroke-related hemiplegia [41]. These variations were observed, probably due to differences in baseline severity, interventions performed in the setting, and the type of resistance focused during applications of various approaches, as reported in studies concentrating on the potential for bimanual training, which is absent in unimanual approaches [38].

These techniques inform clinical implications for children with hemiplegic CP, where mCIMT may be utilized for improving strength and independence during ADLs for unimanual impairments, as supported by previous studies [25,32]. Additionally, for the incorporation of bimanual coordination, HABIT/BIT serves as an alternative that is applied without resistance, leading to social participation [27,29]. Moreover, for resource-constrained settings, mirror therapy provides an adjunctive option for the management of spasticity [23,33]. Furthermore, in infants, mCIMT followed by BIT [30] or technology-enhanced variants [31], which reflect as hybrid models, could enhance facilitation of neuroplasticity [35]. Hence, to upgrade the effectiveness of these approaches, physical therapists should incorporate counselling of parents to reduce fatigue and sustain benefits by assessing MACS levels and family adherence upfront, ultimately enhancing quality of life [30].

Strengths and limitations

The current review executed a comprehensive literature search across multiple databases to synthesize findings reporting comparative effectiveness between mCIMT, HABIT, and mirror therapy approaches from high-quality studies while adhering to the PRISMA-ScR guidelines. However, the review demonstrates certain limitations, which consist of the absence of meta-analysis, variation in intervention protocols, and exclusion of non-English or pre-2020 studies, thereby omitting foundational evidence. Moreover, the studies involved small samples and short follow-ups, which limit outcomes during long-term trajectories and severe impairments.

Recommendations for future research

Future studies should involve standardized protocol regimens, prioritize large-scale, multicenter RCTs with comparisons between mCIMT, HABIT, and mirror therapy, and longer follow-ups. Moreover, studies incorporating control groups should be conducted for comparative effects, along with subgroup analyses by age, lesion type, and socioeconomic factors. Additionally, there is a necessity for studies with hybrid or technology-augmented interventions. These strategies could enhance outcomes mainly in low-resource settings.

## Conclusions

This scoping review demonstrated that mCIMT, HABIT/BIT, and mirror therapy consistently enhance upper extremity function by facilitating neuroplasticity, with mCIMT excelling in unimanual gains, HABIT/BIT in bimanual coordination and participation, and mirror therapy in dexterity and spasticity reduction particularly as an adjunct while exhibiting no adverse effects and short-term sustainability up to six months, though variations in intervention protocols and absence of direct comparisons limit information regarding effectiveness of these approaches. Hence, to enhance independence and quality of life in children with CP, these findings highlight the need for personalized, hybrid interventions like sequenced mCIMT-BIT or technology-augmented regimens across various settings. Moreover, future RCTs with longer follow-ups and standardized protocols are essential to establish evidence-based guidelines, ensuring equitable early access that transforms lifelong trajectories for affected children and families.
